# A comparison of palliative care and rapid emergency screening (P-CaRES) tool, broad and narrow criteria, and surprise questions to predict survival of older emergency department patients

**DOI:** 10.1186/s12904-023-01205-5

**Published:** 2023-06-27

**Authors:** Siripan Koyavatin, Shan Woo Liu, Jiraporn Sri-on

**Affiliations:** 1 Emergency department, Vajira Hospital, Navamindradhiraj University, Bangkok, Thailand; 2 Emergency department, Massachusetts General Hospital, Boston, USA; 3 Geriatric Emergency Medicine Unit. The Department of Emergency Medicine, Vajira Hospital, Navamindradhiraj University, 681 Samsen road. Dusit, Bangkok, 10130 Thailand

**Keywords:** Palliative Care and Rapid Emergency Screening (P-CaRES) tool, Broad and narrow criteria, Surprise questions, Emergency department

## Abstract

**Background:**

Palliative care is a form of medical care designed to enhance the quality of life of patients with life-threatening conditions. This study was conducted to compare the accuracy of predicted survival the 1 and 3-month survival rate of Broad and narrow criteria, Surprise questions (SQ), and Palliative Care and Rapid Emergency Screening (P-CaRES) after admission to the emergency department (ED).

**Methods:**

This prospective cohort study was conducted at an urban teaching hospital in Thailand. Patients aged ≥ 65 years admitted to the ED were classified according to their emergency severity index (ESI) (Level: 1–3). We collected data on SQ, P-CaRES, and broad and narrow criteria. A survival data of participants were collected at 1 and 3 months after admission to the ED. The survival rate was calculated using the Kaplan–Meier and log-rank tests.

**Results:**

A total of 269 patients completed the study. P-CaRES positive and P-CaRES negative patients had 1-month survival rates of 81% and 94.8%, respectively (P = 0.37), and at 3-month survival rates of 70.7% and 90.1%, respectively (P < 0.001). SQ (not surprised) had a 1-month survival rate of 79.3%, while SQ (surprised) had a 97% survival rate (P = 0.01), and SQ (not surprised) had a 75.4% survival rate at 3-months, while SQ (surprised) had a 96.3% survival rate (P = 0.01). Broad and narrow criteria that were positive and negative had 1-month survival rates of 88.1% and 92.5%, respectively (P = 0.71), while those that were positive and negative had 3-month survival rates of 78.6% and 87.2%, respectively (P = 0.19). The hazard ratio (HR) of SQ (not surprised) at 1 month was 3.22( 95%CI:1.16–8.89). The HR at 3 months of P-CaRES (positive) was 3.31 with a 95% confidence interval (CI): 1.74 − 6.27, while the HR for SQ (not surprise) was 7.33, 95% CI: 3.03–19.79; however, broad and narrow criteria had an HR of 1.78, 95% CI:0.84–3.77.

**Conclusions:**

Among older adults who visited the ED, the SQ were good prognosis tools for predicting 1 and 3-month survival, and P-CaRES were good prognostic tools for predicting 3-month survival.

## Introduction

In 2018, it was reported that out of more than 40 million people worldwide that should have received palliative care, only 14% received it [1]. Palliative care is defined by the World Health Organization [1] as the medical care provided to enhance the quality of life for patients with life-threatening diseases. End-of-life patients are those who are experiencing the terminal stages of a disease and cannot be cured. In the hospital, patients with end of life conditions should be enrolled in palliative care programs as soon as possible during their admission to maximize their benefit to the patient, increases satisfaction of patients and the patient benefit in addition to hospital perspective of benefit such as decreased cost and hospital revisits within 30 days. Ranganathan et al. [2] reported that patients receiving palliative care after discharge from the ED had a lower 30-day readmission rate. Morrison et al. [3] also showed that patients in palliative care programs had significantly reduced hospital costs. Moreover, palliative care reduces the usage of life-saving equipment.

In 2013, the American College of Emergency Physicians (ACEP) realized the importance of palliative care and established a palliative care project in emergency departments (EDs) to initiate palliative care earlier in a patient’s hospitalization [4]. Beynon et al. [5] performed a retrospective study to examine whether patients who had died after a recent ED visit had previously been in a palliative care plan; their results showed that only a few patients had received palliative care plans. Importantly, the dearth of palliative care in EDs maybe because it requires the expedited diagnosis of terminally ill patients who could benefit from palliative care in increasingly crowded ED [6]. Usually, ED staff may not have the time to identify patients who might benefit from palliative care or end-of-life plans, and as a result, a quick-screening tool may be useful in implementing palliative care in the ED. According to Nicola et al. [7], the “surprise question (SQ)” accurately predicted mortality in 74.8% of cases. Ouchi et al. studied EDs and found that SQ had a sensitivity of 77% and specificity of 56% for predicting 12-month mortality [8]. Similarly, “Broad and narrow criteria” have been used in EDs. Older adults who have two or more co-morbidities as determined by the Charlson Index are considered “broad.“ Patients that met the criteria for the ‘narrow’ were those who experienced a significant level of physical symptoms [5]. A study measured multi-morbidity using the Charlson Comorbidity Index (CCI), one of the Broad and narrow criteria for quantifying changes in various time windows [9], and used survival models to assess the relationship between CCI changes and mortality. The study compared the mortality rate between the patients that had a change in CCI and those that did not and found an odds ratio of 8.8 (95% confidence interval (CI): 7.5–10.4). Likewise, Palliative Care and Rapid Emergency Screening Tool (P-CaRES) has been used to predict survival in EDs [10, 11]. One prospective study examined whether P-CaRES and the Palliative Performance Scale (PPS) can be used to predict 6 months survival rate after admission from the ED. In this study, the hazard ratio for patients who tested positive for P-CaRES was 4.1 times higher than it was for patients who tested negative for P-CaRES [95% CI: 2.05–8.54], and 51.2% of these patients passed away within six months after being discharged from the hospital [11]. One systematic review of 35 studies to identify patient with unmet palliative care need in the ED found that SQ was the most screening tool used follow by P-CaRES. The study showed that median sensitivity of SQ was 63%(IQR 38-78%) and specificity was 75% (IQR 88–95%) [12].

Care conversations are often guided by prognostic indicators and the context in which screening tools might be useful. Providing palliative care early can not only improve patient quality of life but also reduce unnecessary hospitalizations. Therefore, the aim of this study was to find the most accurate tool for finding end-of-life older patients with chronic diseased who are expected to die within one or three months by comparing between broad and narrow criteria, SQ, and P-CaRES after admission to the ED.

## Materials and methods

This prospective cohort study was performed at one urban teaching hospital in Thailand. The inclusion criteria were patients aged ≥ 65 years admitted to the ED and classified according to the emergency severity index (ESI) levels 1–3 from November 1, 2021, to July 31, 2022. The recruitment time was from 8.00 to 16.00 on weekdays. The exclusion criteria were as follows: patients diagnosed with psychiatric disorders, those who had cardiac arrest before visiting the ED or in the ED, patients who had COVID-19 infection and those who were unable or unwilling to participate in the study. The study was approved by our hospital institutional review board (IRB). This study received funding from Navamindradhiraj University research fund.

### Data collection process

A researcher assistant (RA) who had a bachelor’s degree in public health and three years of experience in geriatric emergency research data collection, and a resident doctor in emergency medicine (PGY-3) informed the participants about the research details at the ED. Before the data collection process, RA and PGY-3 were trained by principle investigator (PI), who had 10 years of experience in caring for geriatric patients in the ED. The recruitment process, every hour, RAs check the emergency department’s computer system to see whether any older individuals are visiting the ED during that time. After the emergency physician evaluated patients and administered treatments or conducted investigations, the recruitment process got underway. Depending on the patient’s state, recruiting began between one and four hours after the patient reached the ED. The average interview time was 15 min per participant. All participants were informed of the confidentiality and consent statements of the study. In cases where a patient was unable to fill the consent form (6-item cognitive screening test [6-ICT] score > 10 [13], indicating severe cognitive impairment), consent was sought from the patient’s first-, second-, or third-degree relatives. Data were collected based on the convenience of RA and PGY-3.

The RA collected baseline demographic data from hospital database, including age, gender, co-morbidity, medications used, underlying diseases, vital signs at triage, and final diagnosis.

The data of activities of daily living (ADL), [14] CCI, [15] clinical frailty scale, [16] triage ESI level [17], vital signs, mode of transportation, ED diagnosis, and ED disposition were collected directly from patients or relatives. The demographic factors utilized to predict mortality rate from the literature were the physiologic score, which comprises vital signs, triage ESI level, functional score, including ADL, and co-morbidity CCI [14, 15, 17–19].

In addition, RA and PGY-3 collected data on SQs (asked emergency physician, who treated the patients of his/her opinions directly), P-CaRES, and broad and narrow criteria. PGY-3 and RA fill out the questionnaire form in the online google sheet data record form.

### Follow-up data collection

The participants were followed up at 1-month and 3-month through telephone calls weather they were alive or had died, hospital database and checking to confirm their date of death in the Thai National Health System database using their identification number (ID).

### Sample size

To our knowledge, no study has compared the accuracy of predicting survival in patients aged ≥ 65 years using the SQ, P-CaRES, and Broad and narrow criteria. Therefore, our study used the proportion from a prior study as the reference to calculate the sample size. Since it has been reported that the survival rate of older adults who visited the ED was 78% at 6 months [11], we estimated the margin of error to be no more than 5% with a 95% confidence level. Based on this calculation, the required number of samples should not be less than 252.

### Outcome measurement

The primary outcome was to compare the 3-month prognostic utility of SQ, P-CaRES, and broad and narrow criteria after admission to the ED.

### Variable definition

- Palliative care: The World Health Organization [1] defines palliative care as the treatment rendered to improve the quality of life of patients with life-threatening conditions.
  Older patients were noted as having “had palliative care plan” if they had a record of a palliative care plan prior to entering the ED or if they had received a decision for palliative care in the ED on the day of their visit. Palliative treatment was provided to this particular patient group.
- End-of-life care: This refers to the care provided to patients who are in the final stages of a disease and cannot be cured.
- Do not resuscitate (DNR) order: This is an order given not to perform cardiopulmonary resuscitation, whether caused by cardiac or respiratory arrest.
- Broad criteria [5]: These individuals have more than two co-morbidities from the CCI or have disorders including multiple sclerosis, dementia, Parkinson’s disease, motor neuron disease, emphysema, congestive heart failure, renal failure, or cancer [9, 15].
- Narrow criteria: These refer to a group of patients who met the broad criteria and presented with physical symptoms, such as pain, breathlessness, weight loss, nausea, vomiting, confusion, anxiety, or the need for additional care from a relative.
  Board and narrow criteria positive were defined as patients who meet the Board’s criteria and presented to the ED with narrow criteria.
- P-CaRES tool: This is a tool that has been validated to identify patients in the ED with unmet palliative care needs. The P-CaRES tool involves two steps: The first step identifies if a patient has a life-limiting condition. While the second step identifies whether the patient has two or more unmet palliative care needs, and if so, palliative care consultation is indicated [10, 11].
  Patients who test positive for P-CaRES have a life-limiting condition in step 1 (such as advanced dementia, advanced CNS disease, advanced cancer, chronic renal failure, advanced COPD, congestive heart failure, class III or IV, end stage liver disease, septic shock, or multi-organ failure in a patient over 65) and have two or more unmet palliative care needs in step 2 (including SQ (not surprised) if this patient died within 12 months, 1 hospital admission or ED visits in the past 6 months with the same condition, visit with difficult to control symptom such as pain, dyspnea, etc, new or worsened complex symptom, long-term care requirements, functional decline, care giver distress).
- Barthel’s Activities of Daily Living (ADL): This criterion is used to assess dependency [14].
- Surprise Question: This is a screening tool used to identify patients nearing the end of life [7]. It does not require clinicians to collect clinical data or use a scoring algorithm, nor does it require clinicians to make a specific estimate of the length of survival. It simply asked whether the respondent would be surprised if the patient died within a specified period.

### Statistical analysis

Patients’ demographic and clinical characteristics were described. Continuous variables are expressed as median (interquartile range, IQR), and categorical variables as percentages. The Wilcoxon rank sum test was used to compare differences in continuous variables between the two groups, while the chi-square test or Fisher’s exact test was used to compare differences in categorical variables. The survival rate was calculated using the Kaplan-Meier and log-rank tests for comparison between the groups. Cox regression was used to determine the factors associated with the mortality rate. Multivariate models were developed by adjusting for covariates (P < 0.1 in the univariate models). The predictive ability of the mortality rate was evaluated using Harrell’s concordance index (C-index). All p values reported were two-sided. Statistical significance was set at p < 0.05. STATA version 15.1 (Stata Corp., College Station, Texas) was used for the analysis.

## Results

**Fig. 1:**
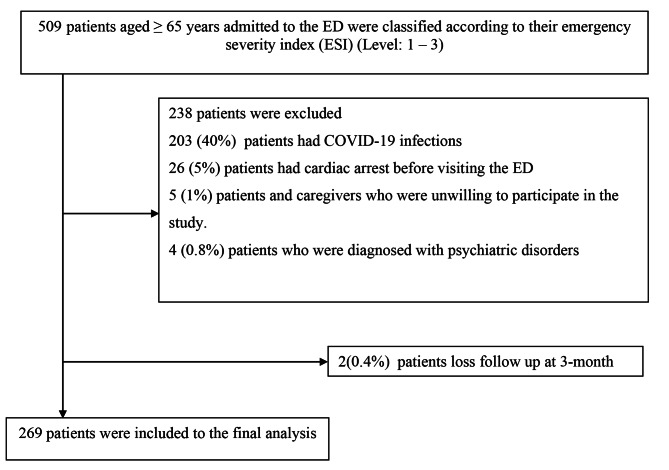
Study flow diagram for subjects enrollment

A total of 509 patients were enrolled in this study. We excluded 203/509 (40%) patients who had covid-19 infections, 26/509 (5%) patients who had cardiac arrest before visiting the ED, 5/509 (1%) patients who were unwilling to participate in the study, and 4/509 (0.8%) patients who were diagnosed with psychiatric disorders. Therefore, 271 patients met the inclusion criteria. However, two (0.4%) patients were lost to follow-up at 3 months, and eventually, only 269 patients were included in the final analysis (Fig. 1).

### Baseline characteristics

The median age of the patients was 76 (IQR: 70–82) years. There was no difference in terms of age between those who were alive versus (vs.) dead at 3-month (alive group, median age = 76 [IQR: 69–82] years vs. death group, median age = 80 [IQR: 70.5–88] years, P = 0.06). One hundred and forty-nine (55.4%) patients were female, and there was no significant difference between the groups. Among the death group, the proportion of patients who had palliative care plans was higher than in the alive group (death group, 11 [27.5%] vs. alive group, 17 [7.4%], P < 0.001). Patients in the death group had more fever cases than those in the alive group (death group, 18 [45%] vs. alive group, 23 [10%], P < 0.001), and the median initial pulse rate (PR) and respiratory rate (RR) of the death group were higher than those of the alive group. Patients in the death group had lower oxygen saturation values than those in the alive group. In addition, the death group had higher CCI scores than the alive group. (Table 1)

**Table 1:** Baseline characteristics of patients

	Total N = 269	Alive N = 229	Death N = 40	P-value
Age (years), median(IQR)	76 (70–82)	76 (69–82)	80 (70.5–88)	0.06
Female, n(%)	149 (55.4)	125 (54.6)	24 (60)	0.53
Had palliative care plan	28 (10.4)	17 (7.4)	11 (27.5)	< 0.001
Fever (BT > 37.5 C)	41 (15.2)	23 (10)	18 (45)	< 0.001
Systolic blood pressure (SBP) mmHg, median(IQR)	143 (122–165)	144 (124–170)	129.5 (109.5–158)	0.03
SBP < 90 mmHg, n(%)	11 (4.1)	9 (3.9)	2 (5)	0.75
Diastolic blood pressure (DBP) (mmHg), median(IQR)	76 (65–86)	78 (66–86)	71 (61–84)	0.06
DBP < 60 mmHg, n(%)	47 (17.5)	37 (16.2)	10 (25)	0.17
Pulse rate (PR), median(IQR)	82 (70–95)	80 (70–92)	96 (82.5-112.5)	< 0.001
PR > 100 bpm, n(%)	52 (19.3)	33 (14.4)	19 (47.5)	< 0.001
Respiratory rate(RR), median(IQR)	20 (18–24)	20 (18–22)	22 (20–30)	< 0.001
RR > 20 bpm, n(%)	48 (17.8)	31 (13.5)	17 (42.5)	< 0.001
O_2_ saturation, median(IQR)	98 (96–100)	98 (97–100)	96 (92.5–99)	< 0.001
O_2_ saturation < 95%, n(%)	36 (13.4)	23 (10)	13 (32.5)	< 0.001
Charlson co-morbidity index (CCI), median(IQR)	5 (4–7)	5 (4–7)	6 (4–8)	0.06
CCI ≥ 7, n(%)	85 (31.6)	66 (28.8)	19 (47.5)	0.02
ADL at baseline, median(IQR)	18 (12–20)	18 (14–20)	13.5 (3–18)	< 0.001
Triage level, n(%)				< 0.001
ESI 1	34 (12.6)	21 (9.2)	13 (32.5)	
ESI 2	155 (57.6)	132 (57.6)	23 (57.5)	
ESI 3	80 (29.7)	76 (33.2)	4 (10)	
Treatment, n(%)				< 0.001
Full life support	233 (86.6)	210 (91.7)	23 (57.5)	
Palliative care	36 (13.4)	19 (8.3)	17 (42.5)	
ED disposition, n(%)				< 0.001
ED observation	36 (13.4)	34 (14.9)	2 (5)	
Discharge home	115 (42.8)	107 (46.7)	8 (20)	
Admit ward	113 (42)	84 (36.7)	29 (72.5)	
Admit ICU	5 (1.9)	4 (1.8)	1 (2.5)	

### The 1-month survival rate

The 1-month survival rates of those with P-CaRES positive and P-CaRES negative were 81%% and 94.8%, respectively (P = 0.37). The 1-month survival rates were 79.3% and 97% for SQ (not surprised) and SQ (surprised), respectively (P = 0.01). The 1-month survival rates were 88.1% and 92.5% for broad and narrow criteria positive and broad and narrow criteria negative, respectively (P = 0.71). The HR of SQ was 3.22 (95%CI 1.16–8.89).

### The 3-month survival rate

**Fig. 2:**
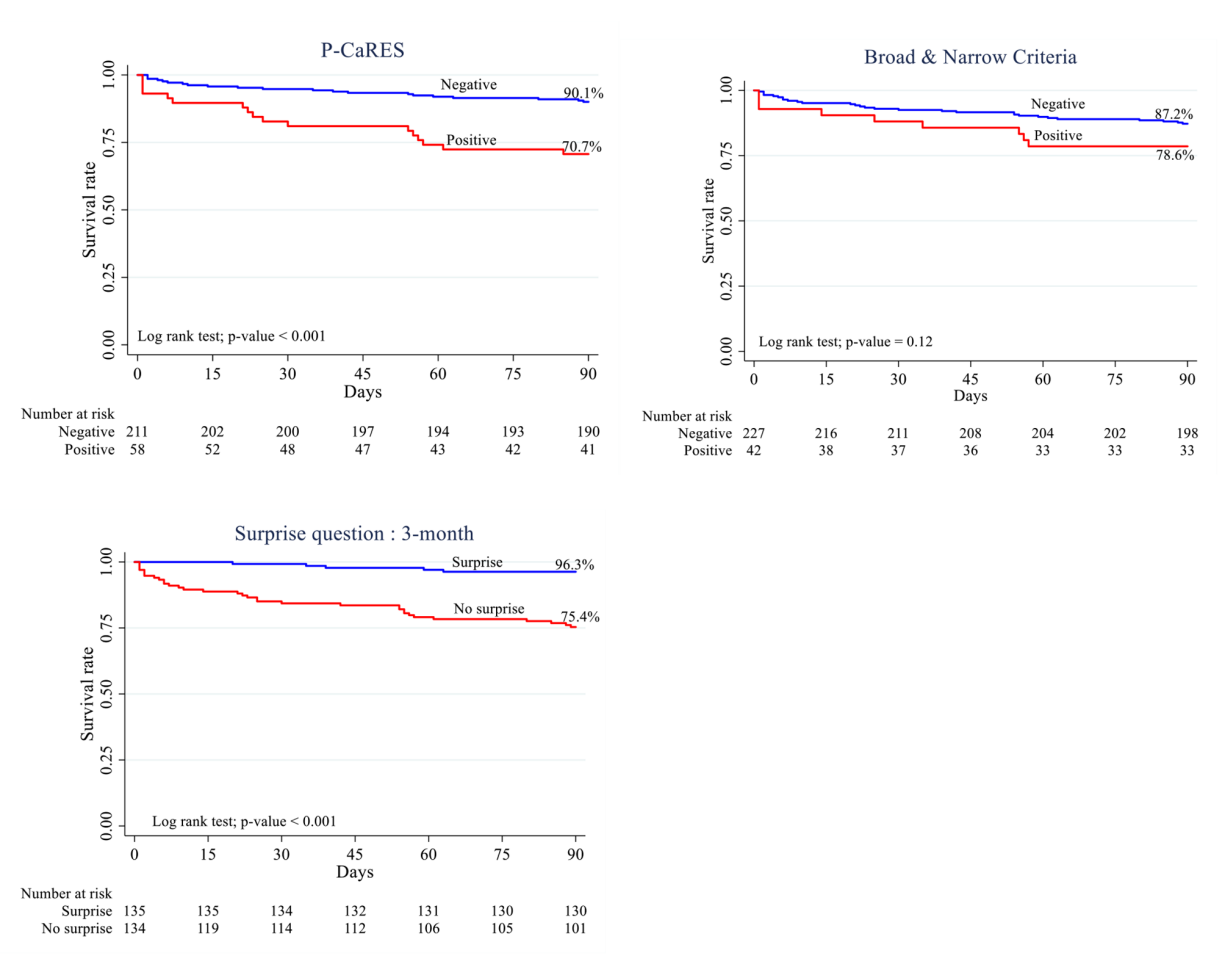
Kaplan-Meier curve for the survival rate of Broad and narrow criteria, P-CaRES, and Surprise question

The 3-month survival rates of those with P-CaRES positive were 70.7%, (P < 0.001). The 3-month survival rates were 75.4% for SQ (not surprised)(P < 0.001). The 3-month survival rates were 78.6% for broad and narrow criteria positive, (P = 0.19). (Table 2; Fig. 2).

**Table 2:** Compare proportion of Broad and narrow criteria, P-CaRES, Surprise question and activities of daily living (ADL) abnormal between alive and death group

	Total N = 269	Alive N = 229	Death N = 40	P-value
Broad & Narrow criteria				0.19
Negative	227 (84.4)	196 (85.6)	31 (77.5)	
Positive	42 (15.6)	33 (14.4)	9 (22.5)	
P-CaRES				< 0.001
Negative	211 (78.4)	188 (82.1)	23 (57.5)	
Positive	58 (21.6)	41 (17.9)	17 (42.5)	
Surprise question				< 0.001
3-month				< 0.001
“No, I would not be surprised”	134 (49.8)	100 (43.7)	34 (85)	
“Yes, I would be surprised”	135 (50.2)	129 (56.3)	6 (15)	
ADL at baseline				< 0.001
< 12	64 (23.8)	45 (19.7)	19 (47.5)	
≥ 12	205 (76.2)	184 (80.4)	21 (52.5)	

Data present n (%), P-value was evaluated by chi-square test

### The predictor of mortality rate

This study showed that P-CaRES positive, SQ (not surprised), and ADL < 12 predicted mortality at 3-month (P-CaRES positive HR: 3.31, 95% CI: 1.74–6.27, and C-index: 0.63, 95% CI: 0.55–0.71; SQ (not surprised) HR: 7.33, 95% CI: 3.03–19.79, and C-index: 0.70, 95% CI: 0.65–0.76; ADL < 12 h: 3.68, 95% CI: 1.95–6.95, and C-index: 0.65, 95% CI: 0.57–0.72); whereas, broad and narrow criteria positive did not (HR: 1.78, 95% CI: 0.84–3.77, and C-index: 0.55; 95% CI: 0.48–0.61). (Table 3)

**Table 3:** Hazard ratio and C-index at 3-month of P-CaRES, Surprise question, and Broad and Narrow criteria

	HR (95%CI)	P-value	C-index (95%CI)
Broad and Narrow criteria: Positive	1.78 (0.84–3.77)	0.13	0.55 (0.48–0.61)
P-CaRES: Positive	3.31 (1.74–6.27)	< 0.001	0.63 (0.55–0.71)
SQ at 3-month: “No, I would not be surprised”	7.73 (3.03–19.79)	< 0.001	0.70 (0.65–0.76)
ADL at baseline < 12	3.68 (1.95–6.95)	< 0.001	0.65 (0.57–0.72)

Cox regression analysis confirmed a significant increase in mortality associated with P-CaRES positivity. (Table 4) From this model, the HR of P-CaRES positive was 2.19 (95% CI: 1.13–4.22). Other variables including initial vital signs at triage: fever (body temperature [BT] > 37.5 °C) (adjusted [a] HR: 3.57; 95% CI: 1.75–7.30), PR ≥ 100 bpm (aHR: 2.14; 95% CI: 1.04–4.45, and RR ≥ 25 bpm (aHR: 2.29; 95% CI: 1.16–4.53) predicted mortality at 3-month. (Table 4)

**Table 4:** The predictor of mortality rate (P-CaRES as primary covariate)

	Univariate		Multivariate	
	HR (95%CI)	P-value	aHR (95%CI)	P-value
Age ≥ 80 years	2.07 (1.09–3.93)	0.03		
Female	1.39 (0.72–2.69)	0.33		
Fever (BT > 37.5 C)	6.59 (3.48–12.49)	< 0.001	3.57 (1.75–7.30)	< 0.001
SBP < 90 mmHg	1.42 (0.34–5.92)	0.63		
DBP < 60 mmHg	1.80 (0.88–3.71)	0.11		
PR ≥ 100 bpm	5.04 (2.66–9.53)	< 0.001	2.14 (1.04–4.45)	0.04
RR ≥ 25 bpm	4.48 (2.36–8.51)	< 0.001	2.29 (1.16–4.53)	0.02
Oxygen Satuation < 95%	4.10 (2.09–8.01)	< 0.001		
CCI ≥ 7	3.68 (1.95–6.96)	< 0.001		
ADL at baseline < 12	2.33 (1.23–4.4)	0.009		
Triage level				
ESI 1	19.9 (4.49–88.23)	< 0.001		
ESI 2	6.19 (1.46–26.25)	0.01		
ESI 3	1	Ref		
Treatment				
Full life support	1	Ref		
Palliative care	7.24 (3.81–13.76)	< 0.001		
Broad and Narrow criteria : Positive	1.78 (0.84–3.77)	0.13		
P-CaRES : Positive	3.31 (1.75–6.28)	< 0.001	2.19 (1.13–4.22)	0.02
			C-index = 0.802	

Cox regression analysis confirmed a significant increase in mortality associated with SQ at 3- month (answered not surprised). (Table 5) The HR of SQ at 3-month (not surprised) was 4.97 (95% CI: 1.90–12.98). Other variables including BT > 37.5 °C (aHR: 3.03, 95% CI: 1.44–6.35), PR ≥ 100/min (aHR: 2.24; 95% CI: 1.10–4.60), and had palliative care plan (aHR: 3.15; 95% CI: 1.58–6.30) predicted mortality at 3-month. (Table 5)

**Table 5:** The predictor of mortality rate [SQ at 3-month (“No, I would not be surprised”) as primary covariate)]

	Univariate		Multivariate	
	HR (95%CI)	P-value	aHR (95%CI)	P-value
Age ≥ 80 years	2.07 (1.09–3.93)	0.03		
Female	1.39 (0.72–2.69)	0.33		
Fever (BT > 37.5 C)	6.59 (3.48–12.49)	< 0.001	3.03 (1.44–6.35)	0.003
SBP < 90 mmHg	1.42 (0.34–5.92)	0.63		
DBP < 60 mmHg	1.8 (0.88–3.71)	0.11		
PR ≥ 100 bpm	5.04 (2.66–9.53)	< 0.001	2.24 (1.10–4.60)	0.03
RR ≥ 25 bpm	4.48 (2.36–8.51)	< 0.001		
Oxygen Satuation < 95%	4.1 (2.09–8.01)	< 0.001		
CCI ≥ 7	3.68 (1.95–6.96)	< 0.001		
ADL at baseline < 12	2.33 (1.23–4.4)	0.009		
Triage level				
ESI 1	19.9 (4.49–88.23)	< 0.001		
ESI 2	6.19 (1.46–26.25)	0.01		
ESI 3	1	Ref		
Treatment				
Full life support	1	Ref	1	Ref.
Palliative care	7.24 (3.81–13.76)	< 0.001	3.15 (1.58–6.30)	0.001
Broad &Narrow : Positive	1.78 (0.84–3.77)	0.13		
SQ 3-month: No surprises	7.74 (3.03–19.79)	< 0.001	4.97 (1.90-12.98)	0.001
			C-index = 0.823	

## Discussion

Our study demonstrates that SQ (“No, I would not be surprised”) predicted mortality at 1 and 3-month and the P-CaRES positive predicted mortality at 3 months. Consistent with our study, Paske et al. [11]. found that 51.2% of the patients who tested positive for P-CaRES died within 6 months after discharge from the hospital. However, they evaluated patients 26 h after admission, unlike our present study, which assessed patients while they stayed in the ED. When evaluating patients in the ED, we can establish care objectives to prevent giving palliative or end-of-life patients needless treatment.

Furthermore, we have validated P-CaRES as a tool for identifying patients with pre-existing conditions and showed that when P-CaRES is combined with other predictors of mortality obtained from triage vital signs, such as body temperature (BT) > 37.5 °C, pulse rate (PR) > 100 bpm, and respiratory rate (RR) > 25 bpm, it may be used as a tool for identifying older adults who would benefit from serious illness conversation, which can help ED physicians identify the appropriate patients who should have palliative care initiated in the ED or soon after.

The P-CaRES tool includes potential terminal diseases that have a high likelihood of accelerating death when combine with aberrant vital signs that could exacerbate the sickness and increase the likelihood of death. The findings of the SQ (“not surprised”) were in accordance with Nicola et al., [7] who found that the SQ was accurate in predicting mortality by 74.8%. On the other hand, a systematic review of a screening tool to determine whether a patient in the emergency department needs palliative care revealed low sensitivity of SQ at 1 month (range 12–43%) while increasing at 12 months (range 57–89%), as well as high specificity at 1 month (range 82–95%) and decreasing at 12 months (range 40–79%) [12]. According to our results, when SQ is combined with other predictors of mortality from the triage and electronic medical record, including BT > 37.5 °C, PR > 100 bpm and had palliative care plan, it showed the greatest c-index for 3-month mortality prediction. It may serve as a variable tool for identifying older adults who would benefit from serious illness conversation and potentially other palliative care services in or after the ED.

In addition, our study evaluated the prediction of survival at 3 months. We expanded the available literature on the association between SQ and 12-month mortality in the ED and other populations. The magnitude of association in our study (HR: 7.73; 95% CI: 3.03–19.79) was higher than in prior studies (odds ratio: 4.4–4.8) [8, 20, 21]. This could be explained by the fact that emergency physicians’ perception of prognosis could be influenced by many factors, such as the clinical experiences, underlying diseases and acute medical conditions [22–25]. It is worth noting that experienced clinicians with more experience were generally more accurate than those with less experience [24, 25]. Another reason might be due to the differences in the acute need for medical care of patients presenting to the ED and the resources for life-sustaining procedures.

Broad and narrow criteria have been used to identify patients with pre-existing conditions who visit the ED with symptoms that may aids ED clinicians to take action on palliative care or have a palliative care consultation; however [5], our study failed to demonstrate that a positive broad and narrow criteria decreased survival at 3 months. The inclusion of board criteria positive that employed CCI—originally designed to predict 10-year survival rather than short-term results—may be the cause of this discovery. CCI was used in this study to predict outcomes at 1 and 3 months, not over a 10-year period [15].

### Clinical implication

We have demonstrated SQ (“no surprised”) predicted survival at 1 and 3 months while that a P-CaRES positive predicted survival at 3 months. When paired with the vital signs, this tool may be helpful for the ED doctor in assisting with discussion of the objective of care with patients or relatives. Future studies should expand the prognosis validity by combining vital signs and disease-specific prognosis tools, such as dementia, COPD, or decompensated heart failure.

### Limitations

This was a single-center study in which subjects were enrolled using convenience sampling depending on the availability of the RA and PGY-3. Further, patients were recruited only from 8.00 to 16.00 on weekdays. Therefore, the sample may have been influenced by selection bias. In addition, the study period overlapped with the COVID-19 pandemic, and the patients under investigation for COVID-19 were not included. Moreover, RA and PGY-3 collected data, and there was no evaluation of the intra-rater reliability. However, studies have shown that the P-CaRES tool is less subjective and has high inter-rater reliability [26, 27]. Finally, the current study was limited by its small sample size; therefore, we could not stratify for each diagnosis.

## Conclusion

From this study, it can be inferred that SQs and P-CaRES may be used to predict survival in patients aged ≥ 65 years admitted to the ED. Further, in addition to their ease of use, employing SQ and P-CaRES in the ED may help ED physicians predict patient survival, plan for better disposition, advocate for patient wishes, and initiate palliative care consultations.

## Data Availability

Datasets used and/or analysed during the current study available from the corresponding author on reasonable request.

## Abbreviations

SQ: Surprise questions
P-CaRES: Palliative Care and Rapid Emergency Screening
ED: emergency department
ESI: Emergency severity index
HR: Hazard ratio
CI: Confidence interval
ACEP: The American College of Emergency Physicians
PPS: The Palliative Performance Scale
RA: Researcher assistant
DNR: Do not resuscitate
PC: Palliative care
CCI: Charlson comorbidity index
6-CIT: 6-item cognitive screening test
IRB: Institutional Review Board
IQR: Interquartile range
EPs: Emergency physicians
C-index: Concordance index
PR: Pulse rate
RR: Respiratory rate
BT: Body temperature
ID: Identification number
